# Animal Models of Nonalcoholic Fatty Liver Disease—A Starter’s Guide

**DOI:** 10.3390/nu9101072

**Published:** 2017-09-27

**Authors:** Mikhaïl A. Van Herck, Luisa Vonghia, Sven M. Francque

**Affiliations:** 1 Laboratory of Experimental Medicine and Pediatrics, Division of Gastroenterology and Hepatology, University of Antwerp, 2610 Antwerp, Belgium; 2 Department of Gastroenterology and Hepatology, Antwerp University Hospital, 2650 Edegem, Belgium; luisa.vonghia@uza.be (L.V.); sven.francque@uza.be (S.M.F.)

**Keywords:** nonalcoholic fatty liver disease, nonalcoholic steatohepatitis, animal model, mouse, rat, hepatocellular carcinoma, high-fat diet, fibrosis

## Abstract

Nonalcoholic fatty liver disease (NAFLD) constitutes a major health concern with the increasing incidence of obesity and diabetes in many Western countries, reaching a prevalence of up to 30% in the general population. Animal models have played a vital role in elucidating the pathophysiological mechanisms of NAFLD and continue to do so. A myriad of different models exists, each with its advantages and disadvantages. This review presents a brief overview of these models with a particular focus on the basic mechanisms and physical, biochemical and histological phenotype. Both nutritional and chemically induced, as well as genetic models are examined, including models combining different approaches.

## 1. Introduction

Nonalcoholic fatty liver disease (NAFLD) is characterized by evidence of hepatic steatosis, in the absence of causes for secondary hepatic fat accumulation. The presence of steatosis and inflammation with hepatocyte injury (ballooning) defines nonalcoholic steatohepatitis (NASH), which may be accompanied by progressive fibrosis [1,2]. NAFLD constitutes a major health concern, as it is in most cases intimately linked with obesity and diabetes, both taking epidemic proportions in many Western countries. In the general population, the prevalence of NAFLD has been estimated between 25% and 30% and between 42% and 70% in patients affected by type 2 diabetes mellitus (DM2) [3,4]. NAFLD constitutes one of the three major causes of cirrhosis and can also be associated with the occurrence of hepatocellular carcinoma (HCC) [5]. Additionally, NAFLD is an independent risk factor for cardiovascular disease [6]. A close relationship has been highlighted between NAFLD and the metabolic syndrome, associating visceral overweight, dyslipidemia, hyperinsulinemia or DM2, and arterial hypertension [2], resulting in the generally accepted conviction that NAFLD is the hepatic manifestation of the metabolic syndrome [7].

The pathogenesis of NASH is complex and implicates a cross talk between various metabolically active sites. According to the “multiple parallel hits hypothesis”, a number of different processes may contribute to liver inflammation. A primary insult is brought about by insulin resistance and fatty acid accumulation, which can affect mitochondrial oxidation of fatty acids causing free radical generation [8]. Secondary hits constitute, among others, oxidative stress, mitochondrial dysfunction and a proinflammatory state [9]. Importantly, NAFLD is no longer considered an exclusively hepatic disease, as multiple other organ systems play a central role in the pathogenesis of liver inflammation [8]. In this setting, two key players are the adipose tissue with an impaired adipokine secretion, which in turn favors a proinflammatory and proadipogenic state [8,10,11], and the gut-liver axis through deregulation of the microbiome [12]. Collectively, these factors are involved in the cascade of inflammation, fibrosis and eventually tumorigenesis. Cytokines, adipokines and cells of the innate and adaptive immune system enable cross-talk between the adipose tissue and the liver and are emerging drivers of the key features of NASH [8,12]. Moreover, evidence for a hepato-cardiovascular axis, in which NAFLD is an independent risk factor for subclinical and clinical cardiovascular disease, is supported by evidence from fundamental and clinical research, underlining the multisystemic impact of NAFLD [6]. Additionally, it has been suggested that NAFLD has its own pathophysiological role in the development of DM2 [13], establishing a multidirectional disease state.

Animal models have played a vital role in elucidating the pathophysiological mechanisms of NAFLD and continue to do so. However, it should be noted that translation of results obtained in an animal model to a human population has repeatedly failed. For example, the phosphodiesterase-4 inhibitor ASP9831 lowered alanine transaminase (ALT) levels, necroinflammation and fibrosis in both an acute hepatitis model and a methionine and choline deficient (MCD) diet NASH model, by selectively inhibiting activated macrophages and Kupffer cells. However, the compound failed to elicit any result in a subsequent clinical trial, in spite of elaborate preclinical investigations and a promising therapeutic target [14]. Other pharmacological interventions have also been very successful in preclinical studies, including the hypolipidemic agent ezetimibe and the antioxidant resveratrol, only to fail in clinical trials [15,16]. This is why it is of utmost importance to choose the best suited animal model relative to the matter under investigation. This review will provide a brief overview of the most frequently used animal models in NAFLD research and serves as a starter’s guide to choose a model, focusing on each model’s major phenotypical features.

## 2. Dietary Models

Mice and rats have been used most frequently in NAFLD modeling and therefore constitute the main focus in this review. The C57BL/6 strain in mice and Wistar and Sprague Dawley strains in rats are generally preferred because of their intrinsic predilection to develop obesity, DM2 and NAFLD [17,18]. Other mammals may also be used, but experience with these models is less substantiated. They may provide interesting insights, however, in specific experimental contexts. For example, the lipid profile in guinea pigs is closer to the human situation compared to mice and rats, which may be of importance when investigating cardiovascular comorbidities in NAFLD [19]. New Zealand white rabbits have a long prepubertal stage and are therefore well suited to investigate pediatric NAFLD [20]. Tree shrews may be an interesting model to investigate nonobese NAFLD, a condition that is more prevalent in Asia, as they tend to develop NAFLD induced by a high-fat diet (HFD) in the absence of obesity [21]. The time of onset, as well as the degree of both NAFLD as accompanying metabolic features are dependent of species, strain, sex, composition of gut microbiota, and the employed dietary intervention [22,23,24].

### 2.1. MCD Diet

The methionine and choline deficient (MCD) diet is one of the best described dietary models for NAFLD. This diet usually has a high sucrose content and moderate fat content (respectively about 40% and 10%). The deficiency in choline and methionine, both essential nutrients, results in impaired β-oxidation and impaired production of very low-density lipoprotein (VLDL) particles [25]. Additionally, choline deficiency brings about an impaired hepatic VLDL secretion, resulting in hepatic fat accumulation, liver cell death, oxidative stress and changes in cytokines and adipokines, but only minor inflammation and fibrosis [26]. The added methionine deficiency brings about a more distinct inflammation and early development of fibrosis (after 8–10 weeks) [25,26,27]. Aspartate transaminase (AST) and ALT levels are significantly increased after 2 weeks of diet and increase progressively [27]. Although the MCD diet results in a rapid onset of the NASH phenotype with lobular inflammation and ballooning (2–8 weeks), the animals do not exhibit any other metabolic features that are seen in human NAFLD, including obesity, peripheral insulin resistance and dyslipidemia. On the contrary, animals fed an MCD diet show significant weight loss (up to 40% in 10 weeks) [25,27,28]. Therefore, this model is generally considered adequate to study the intrahepatic events in relation to NASH and the pharmacological treatment of NASH, but is regarded as inadequate to study the multisystemic disease entity, that is NALFD, in all its aspects [29].

### 2.2. Choline-Deficient L-Amino Acid-defined Diet

The semisynthetic choline-deficient L-amino acid-defined (CDAA) diet is similar to the MCD diet due to their shared deficiency in choline. However, in the CDAA diet proteins are substituted with an equivalent and corresponding mixture of L-amino acids [30]. Animals fed a CDAA diet develop the same or perhaps a slightly more severe degree of NASH, as well as a larger increase in ALT levels, albeit on a marginally longer time frame. After 20–22 weeks, a significant amount of fibrosis is observed [31,32]. Although they do not experience the weight loss observed with the MCD diet, the metabolic features of human NAFLD still fail to appear when used in the same time frame as the MCD diet [25,33]. Nonetheless, after 22 weeks, mice fed a CDAA diet show a significant increase in body weight, plasma triglyceride and total cholesterol levels and HOMA-IR (homeostatic model assessment insulin resistance), suggesting an increased insulin resistance [32]. Moreover, the CDAA diet can be combined with an HFD, establishing an L-amino acid-defined, high-fat diet that brings about a rapidly developing NASH with fibrosis (6–9 weeks), in the absence of substantial weight loss. However, the addition of a high-fat component still fails to elicit features of the metabolic syndrome [34].

### 2.3. Atherogenic Diet

The atherogenic (Ath) diet has proven its usefulness as an animal model of atherosclerosis. The diet contains a relatively high dose of cholesterol (1–1.25%) and cholic acid (0.5%) [35]. The presence of cholic acid promotes cholesterol and fat absorption and suppresses conversion of cholesterol to bile acids, which reduces removal of cholesterol and increases cholesterol levels, particularly non-HDL (high density lipoprotein) cholesterol, thus promoting the development of atherosclerosis [36]. Moreover, the Ath diet induces steatosis (after 6 weeks), inflammation (after 6 weeks), hepatocellular ballooning (after 24 weeks), and fibrosis (after 24 weeks). Additionally, the investigated animals show increased levels of ALT, total cholesterol and, to a lesser extent, triglycerides after 6 weeks [35,37]. However, the Ath diet by itself does not induce weight gain, nor significant insulin resistance. Furthermore, epididymal fat pads, which are generally used as an experimental substitute for human visceral adipose tissue in rodents, appear to be smaller compared to animals fed standard chow (SC). As visceral adipose tissue plays an unmistakable role in NASH and other obesity-related conditions, including cardiovascular disease and DM2, this should certainly be considered a shortcoming [38]. Additionally, glucose and insulin tolerance testing seem to suggest better insulin sensitivity [35]. The addition of a high-fat component (60% cocoa butter) to the Ath diet can ensure hepatic insulin resistance and further accelerate the progression to NASH [35]. 

### 2.4. Fructose

Fructose is a monosaccharide, primarily metabolized in the liver [39]. Excessive intake, as is the case in the USA due to a high consumption of corn syrup, has been linked to the development and increased severity of NAFLD by exacerbating fat deposition, inflammation, oxidative stress, insulin resistance and possibly fibrosis [40]. In both rats and mice, fructose-supplemented drinking water results in simple steatosis after 8 weeks, without features of NASH, and induces a significant increase in body weight, and plasma triglyceride and glucose levels [41,42]. Additionally, intestinal bacterial overgrowth is observed after 8 weeks of treatment, which is followed by increased endotoxin levels in the portal blood and activation of Kupffer cells [42]. Interestingly, weight gain, along with the development of extensive abdominal fat stores, is not necessarily predictive of hepatic steatosis: it has been shown that fructose induces greater hepatic fat accumulation than glucose and sucrose, in spite of a pronounced difference in weight gain in favor of glucose and sucrose [43]. 

### 2.5. High-Fat Diet

This group of diets encompasses multiple regimens with fat contents varying between 45 and 75 kcal% [44]. The excess supply of free fatty acids, directly via intake and via increased lipolysis, brings about triglyceride accumulation in the liver [26]. Interestingly, steatosis develops after 1–2 weeks, but diminishes subsequently, only to reappear after 6–12 weeks [26,45]. Although NASH usually develops after 12 weeks, the observed steatosis and inflammation are substantially less pronounced than is the case in MCD diet-fed animals [28]. The HFD brings about a phenotype similar to the human disease, characterized by obesity (after 10 weeks), insulin resistance (hyperinsulinemia after 10 weeks and glucose intolerance after 12 weeks) and hyperlipidemia (after 10 weeks) [46]. ALT and AST levels are significantly increased after 34–36 weeks. However, this diet induces only minimal fibrosis after extended exposure (36–50 weeks) [46,47]. As was mentioned above, it should be noted that the time of onset and degree of both the metabolic features and NAFLD is dependent of species, strain, sex and composition of gut microbiota, as well as dietary fat content.

When comparing the type of fat used, mice fed a standard HFD show a greater increase in body weight than mice fed trans fats (after 6–8 weeks) [48,49]. Nevertheless, steatosis is more pronounced in mice fed trans fats and ALT levels are higher (after 8–16 weeks) [48,49]. A significantly decreased insulin sensitivity develops after 4 weeks in mice fed trans fats compared to a standard HFD [49]. No differences are observed in levels of cholesterol, nor triglycerides after up to 8 weeks [49]. In rats, after 13 weeks trans fats seem to induce more steatosis than a standard lard-based HFD and this is accompanied by a more pronounced insulin resistance and more disturbed lipid profile. However, no differences are observed in ALT levels between both diets [50].

### 2.6. Variations on the High-Fat Diet

Due to the aggravating effects of fructose on the glucose and lipid metabolism, resulting in increases in visceral adipose tissue deposition, hepatic triglyceride accumulation and insulin resistance, the combined use with an HFD has been proposed [28]. As is the case for the HFD, a myriad of regimens is being used, varying in fat composition and content. Moreover, fructose can be added to the drinking water while animals are being fed an HFD or it can be incorporated in the diet. Sellmann et al. compared a high-fat, high-fructose diet (HFHFD) to an HFD and fructose-containing drinking water alone. After 8 weeks, mice fed the HFHFD showed greater weight gain and more pronounced steatosis compared to the other two groups. After 16 weeks, the HFHFD group showed signs of hepatic inflammation, which was not observed in the other groups. However, no difference in ALT was apparent [51]. Similar results are observed when using a high-fat, high-sucrose diet (HFHSD), with an additional difference in ALT levels in the HFHSD group compared to both mice fed SC and an HFD after 15 weeks [52]. In rats, an HFHFD induces significantly higher plasma triglycerides, higher ALT levels and more steatosis after 2 weeks of treatment compared to an HFD [53].

One of the best described diets is the American Lifestyle Induced Obesity Syndrome (ALIOS) diet, which utilizes a combination of an HFD (45 kcal%) rich in trans fats (30% of fat content) and fructose-containing drinking water [54]. In mice, after 16 weeks this diet induces substantial steatosis with necroinflammatory changes and increased ALT levels. However, no difference is observed in degree of steatosis, nor in ALT levels compared to a high-trans fats diet without the added fructose, in spite of a significantly higher body weight, increased food intake and decreased insulin sensitivity in the ALIOS group [54]. Interestingly, another feature of this model is the promotion of inactivity through removal of the cage racks. Although there is some evidence that experimental exercise can prevent steatosis in HFD-fed rats, the added value of this strategy has not yet been formally proven [54]. 

Yet another variation on the HFD can be achieved by adding a clinically relevant dose of cholesterol to the diet. Charlton et al. investigated a diet with a content of 40 kcal% fat (of which 12% saturated fat) and 2% cholesterol (HFHCD), by comparing it to a regular HFD with a 60 kcal% fat content. All groups received fructose-containing drinking water. At 26 weeks, there was no difference in insulin resistance or body weight between both groups. However, significantly higher levels of AST and cholesterol were present in the HFHCD group. Although the degree of steatosis was similar in both groups, the HFHCD induced significantly more lobular inflammation and ballooning, eliciting a clear picture of NASH. Additionally, some degree of fibrosis was observed, albeit rather mild [55]. Other groups used a similar diet called the Amylin liver NASH model (AMLN), containing 40 kcal% fat (of these 18% trans fats), 20% fructose and 2% cholesterol, thus incorporating fructose in the food, and established similar results [48,56,57].

## 3. Chemical Models

### 3.1. Streptozotocin

Streptozotocin-induced diabetes is a well-known experimental model of DM2 and is achieved by the administration (intraperitoneal or subcutaneous) of a low dose of streptozotocin shortly after birth, which results in a chemical inflammation and destruction of the pancreatic islets. When this approach is combined with an HFD, it can be used as a model for NAFLD [58,59]. In mice, HFD feeding starting at 4 weeks of age and following neonatal streptozotocin administration results in simple steatosis at 6 weeks of age, NASH with inflammatory foci and ballooning at 8 weeks and progressive pericellular fibrosis starting at 8–12 weeks. Starting at 6 weeks of age, the mice show elevated transaminases and fasting glycemia. Additionally, at 20 weeks of age, mice show the presence of multiple hepatocellular carcinomas [59].

An alternative approach is to administer the streptozotocin in a later stage, i.e., after having started the HFD. Lo et al. used a 20-week HFD combined with a streptozotocin-induced DM2 after 16 weeks. Although elevated when compared to mice fed SC, no significant differences in transaminase levels, degree of steatosis or degree of inflammation were observed between mice that received the complete regimen and mice that were exclusively fed an HFD. However, there was significantly more fibrosis in both the central vein and portal tract areas, as well as in the perisinusoidal space, in the streptozotocin-treated group. Remarkably, streptozotocin-treated mice weighed less than HFD-fed mice and, quite logically, insulin levels were lower [58].

### 3.2. Carbon Tetrachloride

Liver damage induced by carbon tetrachloride (CCl_4_) is a well-established general model for liver fibrosis. CCl_4_ induces an oxidative stress response in the liver, which leads to the accumulation of toxic lipid and protein peroxidation products and to a strong necrotic response. In mice, biweekly peritoneal injection of CCl_4_ brings about extensive liver damage with degenerated and ballooned, necrotic hepatocytes, as well as a mild mononuclear cell infiltration and features of macro- and microsteatosis in the affected areas. Transaminase and triglyceride levels are substantially higher compared to those of control animals (injected with vehicle only) [60,61]. Most importantly, CCl_4_ induces a dose-dependent fibrosis that regresses after discontinuing CCl_4_ administration [62]. When used in isolation, CCl_4_ induces fibrosis, but no obesity, nor insulin resistance, and it is no NAFLD model by itself, which is why it is frequently combined with dietary models when modelling NALFD. In this setting, CCl_4_ potentiates the effects of an HFD towards the development of NASH and fibrosis [2,63]. Kubota et al. developed a combined model of HFD feeding and CCl_4_ administration. They demonstrated that, in contrast to mice exclusively fed an HFD, multiple (8 times over 4 weeks) administration of CCl_4_ to HFD-fed mice induced not only steatosis, but also recruitment of inflammatory cells, hepatocellular ballooning, centrolobular fibrosis, both pericellular and perisinusoidal, hypertriglyceridemia and significantly increased transaminase levels after 12 weeks. Additionally, the authors describe a progressive worsening of the histological features with an increasing experimental duration and number of CCl_4_ administrations. However, the mean body weight was significantly lower in the HFD + CCl_4_ group compared to the HFD group and did not differ from the mean body weight of the control group. Moreover, total cholesterol and glucose levels were lower in the HFD + CCl_4_ group compared to both the control group and the HFD group [64]. Similar results were obtained in an eight-week rat model by another group [65]. Analogous to the combination of HFD and CCl_4_, thioacetamide (TAA) can also be used [66], as well as the combination of a different diet, such as a CDAA diet, with CCl_4_ [67].

### 3.3. Diethylnitrosamine 

Diethylnitrosamine (DEN) is a known carcinogen that causes significant oxidant stress and DNA mutations, potentiates lipotoxicity, accelerates progression of fibrosis and cirrhosis and has long been used to model HCC [68,69]. In combination with adequate dietary measures, this model can be used to study the development of HCC in NAFLD. Thompson et al. used an HFD murine model in combination with a one-time intraperitoneal administration of DEN at 21–25 days of age. At 42 weeks. HFD-fed, DEN-treated mice exhibited a NASH phenotype in conjunction with HCC in 8 out of 9 animals, compared to 6 out of 10 SC-fed, DEN-treated mice and 4 out of 10 HFD-fed, vehicle-treated mice. Furthermore, HFD-fed, DEN-treated mice showed a significantly higher weight and ALT levels compared to the control animals [70]. Another possible combination is with an MCD diet. Toriguchi et al. used a 16-week MCD diet murine model in combination with a one-time intraperitoneal administration of DEN at 10 days of age. At the end of the experiment, all MCD diet-fed, DEN-treated mice exhibited multiple HCCs, while SC-fed, DEN-treated mice only did so infrequently [71]. The question remains whether this model is representative of NAFLD-related HCC, as the carcinogenic stimulus, in fact, is not NAFLD.

## 4. Genetic Models

### 4.1. DM2 Models

Leptin is an adipokine produced by white adipose tissue that exerts a marked anorexic effect through the hypothalamus. *Lep^ob^*/*Lep^ob^* (ob/ob) mice display a spontaneous mutation in the leptin gene, which renders them leptin-deficient. Ob/ob mice are hyperphagic, inactive, extremely obese, and display hyperglycemia, insulin resistance (from as early as 3–4 weeks old), and hyperinsulinemia [72]. At as soon as 12 weeks of age, mice show mild to severe steatosis, but even after 20 weeks, ballooning and lobular inflammation remain absent [57,73]. It is generally accepted that the genetic mutation alone is not sufficient for the development of NASH and a second stimulus is needed in the form of a chemical challenge (including lipopolysaccharide, CCL_4_ and TAA administration) or dietary measures (including MCD diet and HFD) [28,62].

Trak-Smayra et al. and Kristiansen et al. compared SC-fed ob/ob mice to ob/ob mice respectively fed a high-calorie diet (HCD), deriving 16 kcal% of its energetic value from fat and a 40 kcal% HFD enriched with fructose and cholesterol, described above (AMLN diet). As early as 4 weeks after starting the diet, body weight and cholesterol levels were higher in the HCD group [73]. Triglyceride and ALT levels were significantly higher in the AMLN group at 12 weeks [57]. Histologically, steatosis was more pronounced in the HCD-fed group compared to the SC-fed group. Although no ballooning was observed, necroinflammation was present in all animals after one month in the HCD-fed group and in 90% of animals in the SC-fed group, albeit in varying degrees [73]. At 20 weeks AMLN-fed mice displayed some ballooning and varying degrees of inflammation, whereas this was not seen in SC-fed ob/ob mice [57]. Although fibrosis is not classically seen in ob/ob mice [74], Trak-Smayra et al. do describe a low-grade fibrosis (perisinusoidal and portal) in nearly all mice (both HCD-fed and SC-fed) after three months [73]. This finding is different from the observations of Kristiansen et al., who found no or only low-grade fibrosis in the SC-fed group, but varying degrees of fibrosis (up to grade 4) in the ALMN-fed group after 12 weeks [57]. Possibly, this is due to the use of different diets with varying fat contents (respectively 16 kcal% and 40 kcal%). When comparing the AMLN-fed ob/ob mice to wild-type C57BL/6 mice fed the same AMLN diet, the ob/ob group shows a greater degree of fibrosis and a tendency to more ballooning and inflammation. No difference was observed in degree of steatosis [57]. It is worth noting that leptin deficiency is a very rare condition in humans and that leptin levels do not correlate well with disease severity in NAFLD patients [75].

*Lepr^db^/Lepr^db^* (db/db) mice possess a natural mutation in the leptin receptor gene, rendering it nonfunctional. As a consequence, these mice exhibit a similar phenotype to that of the ob/ob mice, though with normal to elevated leptin levels [76]. An analogous mutation exists in rats and is depicted as *Lepr^fa^/Lepr^fa^* (fa/fa, also known as Zucker rats). These rats show similar phenotypes to both ob/ob and db/db mice, as they spontaneously develop severe obesity, insulin resistance and steatosis and are hyperleptinemic, hyperphagic and inactive. Both db/db mice and fa/fa rats do not spontaneously develop NASH and an additional stimulus is required, as is the case with ob/ob mice [28]. Another genetic DM2 model involves a mutation in the *Alms1* gene (foz/foz), involved in the hypothalamic control of satiety. This mutation leads to hyperphagy, increased body weight, DM2, and, when combined with an HFD, NASH with fibrosis after 20–24 weeks [44,77]. Sterol regulatory element-binding protein (SREBP)-1c transgenic mice develop severe insulin resistance and NASH with perivenular and pericellular fibrosis, but exhibit decreased adipose tissue mass [25].

### 4.2. Atherosclerosis Models

Apolipoprotein deficient (*ApoE*^−/−^) mice and low density lipoprotein receptor deficient (*Ldlr*^−/−^) mice are predisposed to develop hypercholesterolemia, atherosclerosis and obesity. When combining these models with an HFD, the animals additionally develop NASH [44], which renders them useful models to explore cardiovascular morbidity and the metabolic syndrome in NASH.

### 4.3. HCC Models

Several specific murine knockout models exist to more specifically study the development of HCC in NAFLD, including mutations regarding phosphatase and tensin homolog (*PTEN*), augmenter of liver regeneration (*ALR*) and melanocortin 4 receptor (*MC4R*) [68]. *PTEN* is a tumor suppressor gene, in the absence of which mice spontaneously develop HCC at 74–78 weeks on a background of NASH [78]. *ALR* knock-out mice exhibit excessive hepatic steatosis as early as 2 weeks after birth, that subsequently regresses to disappear at 8 weeks. Conversely, at 8 weeks their livers show inflammation with hepatocellular necrosis and HCCs are found in 60% of the animals at 1 year after birth [79]. *MC4R* mutations are one of the most common monogenetic causes of obesity in humans [68]. In HFD-fed mice, this mutation elicits a clear NASH phenotype with ballooning, inflammation and pericellular fibrosis, which is associated to obesity, insulin resistance and dyslipidemia at 20 weeks and HCC at 1 year [80]. Still other genetic models exist, but fall beyond the scope of this review.

## 5. Conclusions

Models based on a nutritional deficiency such as the MCD and CDAA diets have long been used and have the advantage of eliciting an unequivocal and severe NASH phenotype in a limited time frame. However, it should be noted that this approach utilizes pathophysiological mechanisms that do not coincide with human NALFD. Moreover, these models fail to induce the metabolic comorbidities that are typically observed in human NAFLD (insulin resistance, dyslipidemia and obesity). Therefore, these models should be used with caution and their use should be limited to clearly defined liver-specific research goals.

The same remark can be made regarding the chemically induced models, although this might be overcome by combining the model with appropriate dietary measures. Additionally, it should be noted that the fibrosis induced by toxic agents, such as CCl_4_, is not pathophysiologically related to the disease that is being modeled, constituting another major shortcoming. Nonetheless, these models might be of interest when focusing on a more advanced NAFLD phenotype such as advanced fibrosis, cirrhosis and HCC.

Genetic models present certain advantages concerning experimental duration and NAFLD severity, while retaining the metabolic features associated with NAFLD. However, it should be noted that these mutations are very rare in humans. Additionally, the use of genetic models will substantially increase the experimental expenditure.

Regarding the HFD models, the extensive collection of variations in fat content, type of fat and possible supplements does not make for an uncomplicated choice. The main advantage of this group of diets is the resemblance to human NAFLD, both pathophysiologically and phenotypically. The main disadvantage remains the extended experimental duration and lesser degree of NAFLD severity, although these issues might be resolved in part by diversifying the diet using mono- or disaccharides, trans fats and cholesterol, which should be considered.

As discussed earlier, insulin resistance is not only a central player in the pathophysiology of NALFD, but NALFD is also a strong predictor of the development of DM2, thus establishing reciprocal disease states. Moreover, the presence of DM2 is an independent risk factor for fibrosis progression to an advanced form, especially when obesity coexists [77]. As the presence of DM2 and obesity constitute independent and synergistic risk factors [77], it might be reasonable to consider them as separate risk groups and therefore study them as such. Both the streptozotocin-induced model as the genetic DM2 models can be used in this specific setting, again recognizing the possible shortcomings in each model.

Another issue that is difficult to address experimentally is HCC, a known complication of NAFLD, for which there is a lack of representative animal models to study the matter in vivo. In standard NALFD models, tumor development is slow and infrequent [68]. The specific HCC models available mostly use a nonphysiological mechanism, including genetic mutations and chemical carcinogens, and are time-consuming and expensive.

Lastly, multiple studies have shown the impact of differential compositions of gut microbiota and alterations in inflammasome function on the development of NAFLD and DM2, independently of obesity. Conversely, in dietary models, the type of diet that is used may influence gut microbiota composition. This should be taken into account when interpreting study results [23,24,33]. 

Seemingly, because of the complex, multidirectional pathophysiology involved in NAFLD, the perfect animal model representing the complete NAFLD spectrum in a workable time frame does not exist. Investigators should always remain aware that substantial differences exist between rodents and humans, as is apparent both anatomically (for example in the differential distribution of adipose tissue [38]) and physiologically (for example in the phenomenon of fasting-induced steatosis in rodents [81]). These limitations should certainly not hinder the use of the models, as long as they are recognized. As translation has repeatedly failed in the past, investigators should have a clear perception of what they are studying and should choose the best-suited animal model relative to their research goal, taking into account NAFLD comorbidities, grade of fibrosis and the possible development of HCC. 
